# Modular and coordinated expression of immune system regulatory and signaling components in the developing and adult nervous system

**DOI:** 10.3389/fncel.2015.00337

**Published:** 2015-08-28

**Authors:** Jimena Monzón-Sandoval, Atahualpa Castillo-Morales, Sean Crampton, Laura McKelvey, Aoife Nolan, Gerard O’Keeffe, Humberto Gutierrez

**Affiliations:** ^1^School of Life Sciences, University of LincolnLincoln, UK; ^2^Department of Biology and Biochemistry, University of BathBath, UK; ^3^Department of Anatomy and Neuroscience, Biosciences Institute, University College CorkCork, Ireland; ^4^Irish Centre for Fetal and Neonatal Translational Research (INFANT), Cork University Maternity HospitalCork, Ireland

**Keywords:** nervous system, gene expression, immune system, microarray, co-expression networks

## Abstract

During development, the nervous system (NS) is assembled and sculpted through a concerted series of neurodevelopmental events orchestrated by a complex genetic programme. While neural-specific gene expression plays a critical part in this process, in recent years, a number of immune-related signaling and regulatory components have also been shown to play key physiological roles in the developing and adult NS. While the involvement of individual immune-related signaling components in neural functions may reflect their ubiquitous character, it may also reflect a much wider, as yet undescribed, genetic network of immune–related molecules acting as an intrinsic component of the neural-specific regulatory machinery that ultimately shapes the NS. In order to gain insights into the scale and wider functional organization of immune-related genetic networks in the NS, we examined the large scale pattern of expression of these genes in the brain. Our results show a highly significant correlated expression and transcriptional clustering among immune-related genes in the developing and adult brain, and this correlation was the highest in the brain when compared to muscle, liver, kidney and endothelial cells. We experimentally tested the regulatory clustering of immune system (IS) genes by using microarray expression profiling in cultures of dissociated neurons stimulated with the pro-inflammatory cytokine TNF-alpha, and found a highly significant enrichment of immune system-related genes among the resulting differentially expressed genes. Our findings strongly suggest a coherent recruitment of entire immune-related genetic regulatory modules by the neural-specific genetic programme that shapes the NS.

## Introduction

The orchestrated execution of the neural-specific genetic-code that governs the development and physiology of the nervous system (NS) is crucial for normal neural function (Lister et al., 2013; Parikshak et al., 2013; Willsey et al., 2013). The importance of the correct execution of neural-specific gene expression for normal neural development and function, is highlighted by the fact that perturbations in the specific genetic networks that constitute this wider genetic code lead to neurodevelopmental and/or adult onset disorders of the NS (Brashear et al., 2014; Helsmoortel et al., 2014).

Intriguingly, disturbances in the immune system (IS) is a common theme in a wide variety of disorders of the NS, ranging from childhood autism to depression (Mitchell and Goldstein, 2014). While this could result from direct immune responses (mainly inflammatory) disrupting the neurodevelopmental programme, neurological disorders triggered by the IS could also result from a potential genetic regulatory overlap between the IS and the NS. In support of this possibility, in recent years, a number of signaling molecules and regulatory components originally described in the IS have been found involved in distinct neural-specific functions including early survival of neuronal precursors, dendritic and axonal growth in developing neurons as well as synaptic remodeling and learning and memory mechanisms both in the developing and adult NS (Gutierrez et al., 2005, 2013; O’Keeffe et al., 2008; Gavaldà et al., 2009; Gutierrez and Davies, 2011; Nolan et al., 2011; Twohig et al., 2011; Carriba et al., 2015; Galenkamp et al., 2015). Furthermore, immune system-related genes have been found statistically overrepresented among highly variable genes expressed during early brain development (Sterner et al., 2012) suggesting a critical role for these genes at key stages of NS development. More broadly, changes in gene family size associated with increased encephalization in mammals have been found enriched in IS related functions prominently expressed in the NS (Castillo-Morales et al., 2014). Taken together these findings suggest a potentially wider involvement of large numbers of immune system-related genes in key aspects of NS development and function.

While the involvement of isolated immune-related signaling components in neural functions may reflect their otherwise ubiquitous character, it may also reflect a much wider, as yet undescribed, genetic network of immune–related molecules acting as an intrinsic component of the neural-specific regulatory machinery that shapes the functional complexity of the NS. In order to gain insights into the scale and wider functional organization of immune-related genetic networks in the developing and adult NS, we examined the large scale pattern of expression of these genes in the developing and adult brain. A complex phenotype is usually the result of an assembly of molecular and genetic components acting in concert (Hartwell et al., 1999). As a result, genes involved in related cellular responses display coordinated pattern of expression reflecting their functional association (Eisen et al., 1998; Saris et al., 2009; Torkamani et al., 2010; Obayashi and Kinoshita, 2011; Zhang et al., 2012; Homouz and Kudlicki, 2013). In the NS, this functional organization of closely coordinated genes also displays a substantial degree of conservation across species (Oldham et al., 2006, 2008). In this study, because of their conspicuous presence and involvement in neural-specific functions, we specifically asked whether immune-related regulatory and signaling components operate in isolation from each other but in close association with neural-specific genes or, alternatively, in closer coordination with other immune-related genes.

By combining human gene expression data analysis, with microarray profiling in dissociated developing neurons, this work provides compelling evidence showing that immune-related genetic networks form an intrinsic part of the wider genetic programme that governs NS development and function. We discuss the implications of these findings and their potential relationship to disorders of the NS.

## Materials and Methods

### Gene Expression Data

We used RNA-seq expression data the from the Allen’s Institute Brainspan database.^1^ Reads per kilobase of transcript per million reads mapped (RPKM)-normalized data in this dataset were summarized to Ensembl Gene IDs, and further normalized against total expression per sample. In order to ensure a homogenous representation of structures at all development points used, we selected a subset of this dataset covering 12 brain regions (A1C, CB, DFC, IPC, ITC, M1C, MFC, OFC, S1C, STC, V1C, VFC) and 20 developmental time points ranging from 12 post conception week through to 40 years (12, 16, 21 and 24 pcw; 4 and 6 months; and 1, 2, 8, 11, 13, 15, 18, 19, 21, 23, 30, 36, 37, 40 years old).

Human microarray expression data for normal brain (GSE13162), muscle (GSE11681), kidney (GSE2004), liver (GSE2004) and aortic endothelium (GSE29903) was obtained from Gene Expression Omnibus,^2^ only samples categorized as “normal”, “control” or “healthy” were considered for analysis. Either RMA or median normalized values were summarized to Ensembl Gene IDs by averaging probe expression as described above. Probes that matched multiple Ensembl Gene IDs were excluded from the analysis.

### Gene Ontology Annotations

The lists of genes annotated to the “immune system process” (IS, Gene ontology ID: GO:0002376) and “Neurological system process” (NS, GO:0050877) were obtained from the GO slim GOA database^3^ through Ensembl Biomart.^4^ Genes annotated to both categories were excluded from all the analysis, so as to not overestimate the relation between IS and NS. For the functional enrichment analysis in “IS process” sub-categories (Table 2), we used the annotation available in Ensembl version 73 and used OBOedit software to assign all children GO categories to their corresponding GO parent.

### Co-Expression and Clustering Analyses

Co-expression analyses were carried out by obtaining the Pearson correlation coefficient across all possible pairs of IS-associated or IS-NS-associated genes in the brain, as well as co-expression within IS genes in other human tissues. Measurements of clustering coefficients and statistical analyses including numerical simulations were carried out using the *R* statistical software package, and particularly *igraph* library.

### Functional Enrichment Analysis

Significant over-representation of IS process genes in our microarray data was measured by contrasting the number of genes annotated to a relevant GO with the expected representations of GO terms and their standard deviations numerically derived from Monte Carlo simulations using at least 10,000 equally-sized random samples of genes from the list of Ensembl Gene IDs covered by the microarray profiling (*n* = 15,888). Benjamini-Hochberg multiple testing adjustments against the number of IS process sub-categories tested (*n* = 21) were carried out. Categories with a resulting adjusted *p*-value < 0.05 were deemed significantly enriched.

### Cell Cultures

The superior cervical ganglia (SCG) of embryonic and postnatal Sprague-Dawley rats (Biological Services Unit, UCC) were dissected out and grown in Dulbecco’s Modified Eagle Medium/F12 (DMEM:F12, Sigma) containing 1% penicillin/streptomycin (Sigma), 1% glutamine (Sigma), 1% N2 (Invitrogen), 2% B27 (Invitrogen) and 10 ng/ml NGF (R&D Systems). Dissociated neurons were plated at a low density on poly-ornithine/laminin-coated 4 well 35 mm tissue culture dishes (Sigma; Greiner Bio-One). TNF-alpha (10 ng/ml, Promokine) was added to the medium and the cells were incubated under a humidified atmosphere containing 5% CO_2_ at 37°C for 24 h.

### Microarray Profiling

After 24 h in culture, medium was removed and the cells were washed twice in PBS and total RNA was isolated using the RNeasy mini extraction kit (Qiagen) according to the manufacturer’s instructions. The samples were run in an AGILENT bioanalyser to check RNA quality/integrity prior to being sent for microarray analysis. Microarray hybridization was outsourced through a commercial provider (Source Biosciences) using the Affymetrix GeneChip Rat 1.0 ST array with a 2 ug RNA analyzed per group. Array images were reduced to intensity values for each probe using Affymetrix MAS 5.0 software. RMA was used to correct background and normalize probe levels (AffyPackage). Probes with expression values lower than the average of negative controls in every sample was removed. Expression values were summarized to Ensembl Gene IDs by averaging probe expression. Finally, gene expression was normalized against the total signal level in each sample.

## Results

### Highly Correlated Expression of Immune System Genes in the Nervous System

In order to characterize the functional interactions of IS genes in the NS; we used gene co-expression network analysis, an approach that has been widely used to gain insights into the functional organization of transcriptomes across tissues, conditions and species (Eisen et al., 1998; Saris et al., 2009; Torkamani et al., 2010; Obayashi and Kinoshita, 2011; Zhang et al., 2012; Homouz and Kudlicki, 2013). We started by asking whether IS genes show a stronger pattern of coordinated activity with genes specifically involved in neural functions relative to the background gene population. To this end, we used gene expression data obtained from the normal developing human brain^5^ and obtained the list of genes annotated to the “Immune system process” and “Neurological system process” from the GO slim GOA database.^6^ In order to focus our study on those genes separately involved in these two processes, we excluded genes simultaneously annotated to both categories. This resulted in a total of 1584 and 1036 genes annotated to “Immune system process” and “Neurological system process”, respectively, for which human expression data was available in the Brainspan dataset.

**Figure 1:**
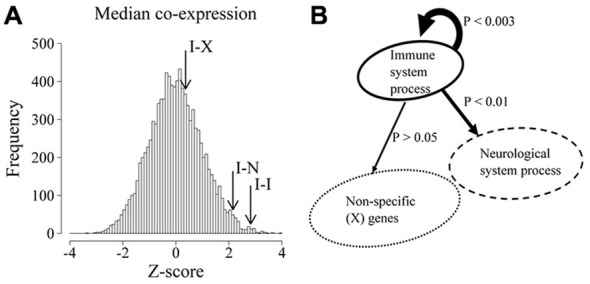
**Immune system (IS) process associated genes are highly co-expressed in the developing and adult brain. (A)** Distribution of median co-expression values of 10,000 equally sized random samples of genes. Co-expression is expressed as *Z* score-transformed median correlation coefficients relative to the expected distribution. Arrows show the observed median co-expression between IS process genes (I-I), IS process and neurological system process genes (I-N) as well as IS process genes and random non-specific genes (I-X). **(B)** Schematic representation of the statistical bias (*p* values) in co-expression between the indicated populations of genes.

To quantify the overall co-expression within and between these groups of genes, we obtained the median Pearson correlation coefficient of all possible pairs of IS and NS genes as well as all possible pairs of IS-IS genes and contrasted these values with the expected median correlation derived from 10,000 equally sized samples of random genes drawn from the background gene population. As shown in Figure 1, the highest level of co-expression in the brain occurs between IS genes and other IS genes (IS-IS interactions, *p* = 0.003) followed in second place by the median co-expression between IS and NS genes (IS-NS interactions, *p* = 0.018). It is noteworthy that NS-NS co-expression is also remarkably high when compared to equally-sized random samples (*Z*-score 4.45, *p* < 0.0001 not shown in figure). By contrast the median correlation between IS genes and any random non-specific genes on the other hand was not significantly different from chance expectations (*p* > 0.1). These results demonstrate that, in the NS, IS genes are more highly co-expressed with other IS genes than expected by chance and that this level of co-expression was also higher than the observed co-expression between IS genes and genes associated with neurological system processes.

### Highly Correlated Expression is not a General Feature of Immune System-Related Genes in Non-Nervous Tissues

If the highly coordinated expression of IS genes observed in nervous tissue was also found outside the NS, it could reflect the ubiquitous modular organization of IS genes and not their particular recruitment by the NS. We tested this hypothesis by analysing independent microarray gene expression data for various human tissues. To this end, we used data obtained with the same microarray platform and derived from human tissues for which at least six biological samples were available. We found five datasets meeting these requirements corresponding to whole brain, liver, aortic endothelium, muscle and kidney (see “Materials and Methods” Section).

**Figure 2:**
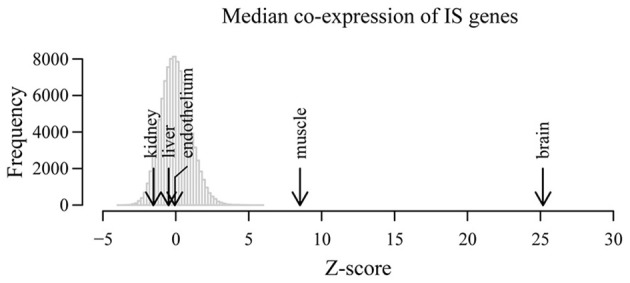
**Correlated expression is not a general feature of immune system-related genes in non-nervous tissues**. Co-expression of IS genes was examined in independent microarray expression data derived from whole brain as well as liver, endothelial cells, kidneys and muscle. Histogram shows the distribution of median co-expression values of 10,000 equally-sized random samples of genes in five different tissues. All expected and observed co-expression values and corresponding distributions were *Z* score-transformed in order to compare across tissues. Arrows show the observed *Z*-score transformed median co-expression between IS process genes in the indicated tissues.

After normalization, we obtained for each tissue the median co-expression between all possible pairs of IS genes and compared these values to the expected median co-expression obtained from 10,000 equally sized random samples of background genes. As shown in Figure 2, the highest level of correlated expression of IS-related genes was found in whole brain (*p* < 10^−16^), further confirming our previously observed high coordination of IS genes in the NS. To a much lesser extent, muscle tissue also displayed a high coordination of IS-related genes. By contrast, liver, kidney and endothelium showed non-significant levels of coexpression when compared with random expectations (*p* > 0.1). These results demonstrate that the observed highly concerted expression of IS-related genes in the NS is not a general feature of the expression pattern of these genes across tissues.

### Immune System-Related Genes Display a High Regulatory Clustering in the Developing and Adult Nervous System

The observed bias in the median correlation of IS genes in the NS could be the result of either a large number of isolated pairs of highly correlated genes or, alternatively, the result of a large highly interconnected network of tightly co-regulated genes.

**Figure 3:**
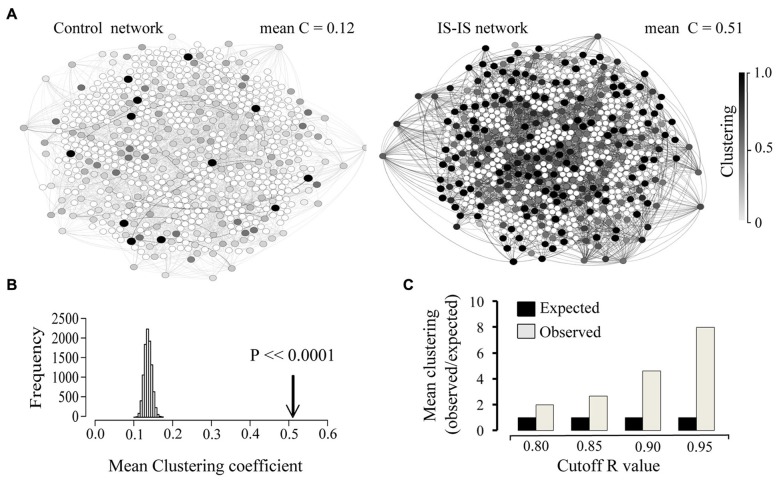
**Immune system genes display a high co-expression clustering in the developing and adult brain. (A)** Network representation of 662 IS genes strongly correlated (edges = *R* > 0.9) with other genes and an equivalent random network with the same size (number of nodes), density (number of edges) and degree distribution (edges per node). Only associations between IS genes are shown (IS-IS links). The clustering coefficient of each node is represented by the corresponding color intensity (white = 0, black = 1). Mean clustering coefficient of each network is indicated. **(B)** Comparison between the observed Clustering (arrow) and the distribution of expected mean clustering coefficients obtained from 10,000 simulated random networks with same size, density and degree distribution as the real network. **(C)** Chart showing the ratio of observed vs. expected clustering coefficient across a range of correlation coefficient value cut-off thresholds.

In order to obtain information on the structure of the involved interactions, we focused on all existing correlations above a given threshold (*R* > 0.9). The resulting map of interactions can be represented as a network, where nodes represent genes and edges represent existing correlations with a coefficient value above 0.9. At this threshold, 662 IS genes were found involved in strong correlations either with other IS genes or any other genes. Given a set number of interactions (edges), the resulting network can display different degrees of clustering between existing nodes. In order to assess the degree of clustering, we obtained the clustering coefficient of each node. This index quantifies, for a given node, the fraction of existing interactions between all immediate neighbors (Watts and Strogatz, 1998). Next we compared the average clustering coefficient of the whole IS-IS network with that of a simulated random network where the number of nodes, number of edges and distribution of edges per node (degree distribution) was the same as the real network. As shown in Figure 3A the observed mean clustering coefficient of the network of highly correlated IS genes was considerably higher than that of the random control network. In order to estimate the significance of this bias, we compared the mean clustering coefficient of IS genes, with the expected mean clustering coefficient derived from 10,000 simulations of equally sized random networks with the same number edges and degree distribution (edges per node). As shown in Figure 3B, the observed clustering coefficient was on average five times higher than the average expected value (*p* < 0.0001). The observed high mean clustering coefficient of IS genes relative to chance expectations holds over a range of correlation cut-off values as shown in Figure 3C, overall demonstrating that IS genes display a significantly high level of clustered co-expression with other IS genes in the NS. It is also worth noting that within the network of highly correlated IS genes, the overall proportion of strong correlations among IS genes (number of IS-IS edges) is also significantly higher than expected by chance relative to the total number of associations involving IS genes (*X*^2^ = 8.912, *p* = 0.0028).

### Coordinated Activation of Large Numbers of Immune System Genes in Primary Cultured Neurons

Taken together, the above results demonstrate that, in the NS, IS-related genes display a stronger overall association with other IS genes than with genes involved in neural-specific functions. In addition, IS genes display a higher average clustering than expected by chance further revealing that IS-related signaling and regulatory components operate under a tightly coordinated pattern of transcriptional regulation in the NS when compared with background genes. Based on these findings, we hypothesized that upon stimulation, IS genes will trigger the simultaneous activation of a large number of other IS-associated genes in isolated neurons.

We tested this prediction using gene expression microarray profiling in dissociated cultures of developing sympathetic neurons derived from the SCG. We chose this neuronal population for three reasons: (1) SCG neuronal cultures offer an experimentally tractable model consisting in >95% neurons from a homogeneous neuronal population, thereby eliminating the confounding influence of highly heterogeneous populations of neurons and glia present in dissociated cultures from other regions of the NS (Orike et al., 2001; Glebova and Ginty, 2005); (2) These neurons have been shown to respond to tumor necrosis factor alpha (TNF-alpha), a cytokine that negatively regulates axonal growth during early postnatal stages (Twohig et al., 2011; Kisiswa et al., 2013; Nolan et al., 2014), thereby offering an ideal opportunity to experimentally assess the effect of an immune-associated cytokine on the global gene expression of dissociated neurons; and (3) While central and peripheral neural tissues share over 99% of their gene expression profiles (LeDoux et al., 2006; Smith et al., 2011), the use of peripheral neurons provides an additional opportunity to test whether the observed regulatory clustering of immune related genes extends to both central and peripheral neurons.

Accordingly, we stimulated cultured neonatal SCG neurons with TNF alpha (10 ng/ml) for 24 h and cells were then collected for total RNA extraction and preparation for gene expression profiling (see “Materials and Methods” Section).

We identified differentially expressed genes based on the rank products method (Hong et al., 2006) and measured the overall response of IS-associated genes by analysing enrichment of IS-associated genes among those genes displaying the highest levels of differential expression (upregulation) upon TNF-alpha stimulation (*p* < 0.05, *n* = 302).

As shown in Table 1, significantly up-regulated genes showed a statistically significant enrichment of IS process-annotated genes. In order to identify subcategories of IS genes particularly over-represented among these genes, we measured enrichment of all gene ontology subcategories associated with IS process relative to the background gene population. This analysis revealed a significant over-representation of genes associated with immune response, leukocyte migration, regulation of IS process, positive regulation of IS process and activation of immune response (Tables 2, 3). These results demonstrate that, in isolated neurons, upon stimulation, IS genes trigger the simultaneous activation of a disproportionally large number of other IS-associated genes when compared to chance expectations.

**Table 1:** **GO enrichment analysis of differentially expressed genes relative to background gene population**.

GO Term ID	Category name	Observed genes	Expected genes	Numeric *p* value
GO:0002376	Immune system process	22	8.9139	<<0.0001

**Table 2:** **GO enrichment analysis within immune system (IS) process genes relative to background gene population**.

GO Term ID	Category name	Observed genes	Expected genes	Numeric *p* value	Adjusted numeric *p* value
GO:0006955	Immune response	22	7.2773	0	0
GO:0050900	Leukocyte migration	9	2.0063	0	0
GO:0002682	Regulation of immune system process	19	8.1524	2.0 × 10^−^^4^	0.001
GO:0002684	Positive regulation of immune system process	13	5.0599	5.0 × 10^−^^4^	0.002
GO:0002253	Activation of immune response	6	2.2173	0.0067	0.022

**Table 3:** **List of IS-associated genes significantly up regulated in response to TNF-alpha stimulation of developing sympathetic neurons**.

Ensembl gene ID	Gene symbol	Description	Immune system process	Immune response process	Leukocyte migration system process	Regulation of immune response of immune	Positive regulation	Activation of immune
ENSRNOG00000007917	Cd46	CD46 molecule, complement regulatory protein		1		1	1	1
ENSRNOG00000011971	C1s	Complement component 1, s subcomponent	1	1		1	1	1
ENSRNOG00000014832	Mapkapk3	Mitogen-activated protein kinase-activated protein kinase 3	1	1		1	1	1
ENSRNOG00000019440	Kcnn4	Potassium intermediate/small conductance calcium-activated channel, subfamily N, member 4				1	1	1
ENSRNOG00000033134	Mef2c	Myocyte enhancer factor 2C		1		1	1	1
ENSRNOG00000033879	Clec7a	C-type lectin domain family 7, member A		1		1	1	1
ENSRNOG00000000239	Ccl7	Chemokine (C-C motif) ligand 7	1	1	1	1	1
ENSRNOG00000004498	Scin	Scinderin				1
ENSRNOG00000008409	Myo1f	Myosin IF	1	1		1
ENSRNOG00000009912	Fgr	FGR proto-oncogene, Src family tyrosine kinase	1	1		1	1
ENSRNOG00000010906	Ccl5	Chemokine (C-C motif) ligand 5	1	1	1	1	1
ENSRNOG00000013794	Rbp1	Retinol binding protein 1, cellular				1
ENSRNOG00000014333	Vcam1	Vascular cell adhesion molecule 1	1		1	1	1
ENSRNOG00000015618	Wnt5a	Wingless-type MMTV integration site family, member 5A		1	1	1	1
ENSRNOG00000016294	Cd4	Cd4 molecule	1	1		1	1
ENSRNOG00000018659	Csf1	Colony stimulating factor 1 (macrophage)	1	1		1
ENSRNOG00000024899	Cxcl13	Chemokine (C-X-C motif) ligand 13	1	1	1	1	1
ENSRNOG00000028015	Pf4	Platelet factor 4	1	1	1	1
ENSRNOG00000032224	Hist2h4	Histone cluster 2, H4				1
ENSRNOG00000008837	Ass1	Argininosuccinate synthase 1	1	1
ENSRNOG00000016535	Ccl22	Chemokine (C-C motif) ligand 22	1	1
ENSRNOG00000022298	Cxcl11	Chemokine (C-X-C motif) ligand 11	1	1
ENSRNOG00000026647	Cxcl16	Chemokine (C-X-C motif) ligand 16	1	1	1
ENSRNOG00000028548	Ccl9	Chemokine (C-C motif) ligand 9	1	1
ENSRNOG00000028768	LOC10091 1495	Guanylate-binding protein 4-like	1	1
ENSRNOG00000031743	Gbp2	Guanylate binding protein 2, interferon-inducible		1
ENSRNOG00000032240	Gbp5	Guanylate binding protein 5	1	1
ENSRNOG00000017197	Pdgfb	Platelet-derived growth factor beta polypeptide	1		1
ENSRNOG00000043451	Spp1	Secreted phosphoprotein 1	1		1
ENSRNOG00000011238	Tiparp	TCDD-inducible poly (ADP-ribose) polymerase	1
ENSRNOG00000019494	Psmb10	Proteasome (prosome, macropain) subunit, beta type 10	1

## Discussion

A wide variety of homeostatic perturbations of the NS including pathogen invasion, endogenous disease and injury, are known to induce an inflammatory response which often involves infiltration of immune cells and activation of resident effectors, such as microglia. While the complex interaction between the immune surveillance machinery and the NS has been the focus of a large number of studies in the past (Ousman and Kubes, 2012; Ransohoff and Brown, 2012), in recent years a growing number of signaling and regulatory components of the IS have emerged as key molecular players in a wide variety of neural-specific functions ranging from early survival of neuronal precursors and dendritic and axonal growth in developing neurons through to synaptic remodeling and learning and memory mechanisms (Gutierrez et al., 2005, 2013; O’Keeffe et al., 2008; Gavaldà et al., 2009; Gutierrez and Davies, 2011; Nolan et al., 2011; Carriba et al., 2015; Galenkamp et al., 2015). Whether these findings reflect a wider and generalized involvement of IS-related regulatory and signaling components in neural-specific mechanisms, is unknown.

By interrogating the wider regulatory organization of IS-associated signaling and regulatory components in the developing and adult NS, in this study we have found that IS genes are more highly co-expressed with other IS genes than expected by chance. In addition, we found that the underlying co-expression network of highly associated IS genes displays a much higher clustering coefficient than expected in networks with equal density and degree distribution. These results reveal a strong underlying regulatory association between large numbers of IS genes operating in the NS.

The recruitment, in the NS, of signaling and regulatory components of the IS could, in principle, simply reflect the ubiquitous character of these regulators, and we would therefore expect them to establish, close but independent functional associations with the wider machinery of neural-specific functions. However, in the NS, IS genes display a stronger association and regulatory clustering with other IS genes than with the wider molecular machinery involved in neural-specific functions suggesting a coherent functional recruitment of entire segments of the IS regulatory machinery by the NS.

We experimentally tested this regulatory clustering in dissociated neurons by conducting microarray gene expression profiling of isolated developing neurons in culture, and found that stimulation with the pro-inflammatory cytokine TNF-alpha triggers the simultaneous activation of a disproportionally large number of other IS-associated genes. The fact that we tested a prediction derived from human expression patterns in experimentally tractable cultured neurons derived from the developing rat, further suggests that the coherent recruitment of IS regulatory clusters is conserved between rodents and humans. While subtle differences at this level may exist between the rodent and human model, this finding is in line with the fact that most basic aspects of extra and intracellular signaling events and their underlying networks of regulatory interactions show a remarkable degree of conservation between murine and human cell models (Shortman and Liu, 2002; Herschkowitz et al., 2007). In addition, the fact that our experimental test was carried out in peripheral neurons further shows that the observed regulatory clustering of immune related genes extends to both central and peripheral NS, an observation otherwise consistent with the highly similar gene expression profiles reported for central and peripheral neurons (LeDoux et al., 2006; Smith et al., 2011).

In addition, by comparing patterns of coordinated expression of IS-related genes outside the NS, including cell types where immune regulators are known to play central roles such as hepatocytes and endothelial cells (Gargalovic et al., 2006), we demonstrate that the highly coherent expression of IS-associated genes observed in the NS is by no means a general feature of these genes outside neural tissues.

Taken together, our results support the notion of a widespread and modular recruitment of IS regulatory and signaling circuits by the NS developmental programme in mammals.

Given the tight regulatory association of large numbers of IS genes in the developing and adult NS, our results raise the possibility of numerous instances of potential interference with neural physiology arising from organismal immune states not directly related with immune surveillance or inflammation in the NS.

Thus, for instance, during pregnancy, maternal infection in the second trimester increases the risk, for affected offspring, of developing psychiatric and neurological disorders such as schizophrenia and autism (Sørensen et al., 2009; Atladóttir et al., 2010; Boksa, 2010). The mechanisms linking maternal inflammation with defective neural development however are unclear. Furthermore, maternal infection in rodents during late gestation results in morphological, electrophysiological and molecular changes in the brains of offspring (Garbett et al., 2012). While this could result from direct immune responses (mainly inflammatory) disrupting the neurodevelopmental programme, our findings suggest that neurological disorders triggered by the IS could also result from the underlying genetic regulatory overlap between the immune and the NS. Interestingly, maternal infection in rodents also triggers changes in proinflammatory cytokine levels in the fetal brain and fetal blood (Garay et al., 2013). Whether these changes can interfere with the establishment of normal developmental patterns in neurons is unknown. However, our results would predict that systemic changes in pro-inflammatory cytokines could potentially trigger concomitant expression changes in IS-related genes in developing neurons leading to measurable alterations in their developmental programme.

## Conclusion

In summary, our results demonstrate that IS genes display a significantly strong level of concerted regulation and transcriptional clustering in the developing and adult brain, supporting the notion of a coherent and widespread recruitment of IS regulatory components by the NS developmental programme in mammals. These results further provide a genetic basis for potential interference with neural functions arising from systemic changes in immune surveillance and inflammatory states.

## Author Contributions

HG and GOK conceived, designed and wrote the manuscript. ACM and JMS carried out the bioinformatics and functional genomics analysis and interpretation of data, SC, LMK and AN carried out the cell culture and microarray profiling experiments.

## Conflict of Interest Statement

The authors declare that the research was conducted in the absence of any commercial or financial relationships that could be construed as a potential conflict of interest.

## Footnotes

^1^http://www.brainspan.org/

^2^http://www.ncbi.nlm.nih.gov/geo/

^3^www.Geneontology.org

^4^http://www.ensembl.org/biomart/

^5^http://www.brainspan.org/

^6^www.Geneontology.org
